# Physical Performance Changes Across Race and Region Among Black and White Older Adults

**DOI:** 10.1001/jamanetworkopen.2026.9937

**Published:** 2026-04-30

**Authors:** Srishti Shrestha, William Windham, Hunter C. Sylvester, Xiaoqian Zhu, Roland J. Thorpe, Priya Palta, A. Richey Sharrett, Anna Kucharska-Newton, Thomas H. Mosley, Michael E. Griswold, B. Gwen Windham

**Affiliations:** 1 Memory Impairment and Neurodegenerative Dementia (MIND) Center, University of Mississippi Medical Center, Jackson; 2 Forrest General Hospital Family Medicine Residency Program, Hattiesburg, Mississippi; 3 Program for Research on Men’s Health, Johns Hopkins Center for Health Disparities Solutions, Johns Hopkins Bloomberg School of Public Health, Baltimore, Maryland; 4 Department of Neurology, University of North Carolina at Chapel Hill; 5 Department of Epidemiology, Johns Hopkins Bloomberg School of Public Health, Baltimore, Maryland; 6 Department of Epidemiology, University of North Carolina at Chapel Hill

## Abstract

**Importance:**

Identifying factors that explain differences in physical performance measures by race and region in the US and whether they are associated with changes over time could inform preventive strategies.

**Objective:**

To examine 10-year changes in physical performance across race and race-region among Black and White older adults and whether differences are explained by demographic, cardiovascular, socioeconomic, and cognitive factors.

**Design, Setting, and Participants:**

The prospective Atherosclerosis Risk in Communities (ARIC) cohort study recruited participants between 1987 and 1989. This study included participants from 4 US communities in North Carolina, Mississippi, Maryland, and Minnesota who underwent physical performance evaluations initiated in 2011 to 2013 (visit 5 examinations) and in 3 subsequent in-person examinations conducted over a 10-year period through 2022. Data were analyzed from January through May 2025.

**Exposure:**

Self-identified race and region.

**Main Outcomes and Measures:**

Physical performance was assessed using the Short Physical Performance Battery (SPPB; score range, 0-12, with higher scores indicating better performance). Multivariable generalized estimating equations estimated cross-sectional differences as well as differences in 10-year changes by race and by race and region, adjusting for demographic, cardiovascular, socioeconomic, and cognitive factors.

**Results:**

Among 5666 participants (mean [SD] age, 75.4 [5.1] years; 3258 [58%] women), 1233 (22%) self-identified their race as Black and 4433 (78%) self-identified their race as White. Adjusting for demographic, cardiovascular, socioeconomic, and cognitive factors explained observed cross-sectional differences in physical performance between Black participants and White participants. However, 10-year SPPB longitudinal declines were steeper for Black participants compared with White participants (absolute difference, −0.80 points; 95% CI, −1.19 to −0.41 points), even after adjusting for all factors that accounted for cross-sectional differences; this finding met the 0.50-point threshold for a clinically meaningful difference. Between-region declines within races also showed notable differences; for example, SPPB declines were steeper for White Maryland participants compared with White North Carolina participants (absolute difference, −1.14 points; 95% CI, −1.45 to −0.83 points), and declines were steeper for Black Mississippi participants compared with Black North Carolina participants (absolute difference, −1.72 points; 95% CI, −2.65 to −0.78 points).

**Conclusions and Relevance:**

In this cohort study of older adults, meaningful racial differences in physical performance declines were not explained by commonly suggested drivers of performance differences. Other unmeasured regional features may explain racial differences in decline in physical performance and warrant investigation.

## Key Points

**Question:**

Do demographic, cardiovascular, socioeconomic, and cognitive factors or geography explain differences in physical performance measures among Black and White older adults?

**Findings:**

In this cohort study of 5666 participants, 10-year decline in physical performance was twice as steep for Black participants compared with White participants, with a clinically meaningful absolute difference, after adjusting for a range of factors. Marked between-regions differences within races were observed; White participants across some states were more different than Black participants compared with White participants within the same state.

**Meaning:**

This study suggests that racial differences in declining physical performance among older adults were not explained by socioeconomic, cardiovascular, or cognitive factors and may be associated with undefined regional exposures.

## Teaser

This cohort study examines 10-year changes in lower-extremity physical performance across race and region among Black and White older adults and assesses whether differences are explained by demographic, cardiovascular, socioeconomic, and cognitive factors.

## Introduction

Poor physical performance, particularly lower extremity function, is associated with adverse outcomes, including poor quality of life, future disability, and higher institutionalization and mortality rates, and imposes a significant personal and public health economic burden.^1,2,3,4^ Studies suggest disparities in mobility disability and physical performance measures among Black people compared with White people and potentially greater functional declines with increasing age among Black adults.^5,6,7^ For example, Black older adults experience higher rates of self-reported transitions to worse disability stages and death and are less likely to maintain successful accommodation to impairments than non-Hispanic White older adults.^8^ Although the reasons are not understood, differences have been attributed to a disproportionate burden of cardiovascular conditions and risk factors,^9,10^ cognitive impairment,^11^ or poor socioeconomic factors,^12^ all of which have been linked with poor physical function.^13,14,15,16,17,18,19^ However, a comprehensive assessment of the association of these factors with objective physical performance measures in diverse populations is lacking.

Prior studies have found that differences in cardiovascular risk factors, cognitive impairment, and socioeconomic status only partly explain racial differences in physical function,^20,21,22^ while others have reported no differences after accounting for these factors.^23,24,25,26,27^ Divergent findings could be due to self-reported vs objective performance metrics, differences in study populations, geographical region–specific factors, or comorbidities and socioeconomic measures.^6,7,20,21,22,24,27,28,29,30,31^ Furthermore, prior studies were mostly cross-sectional; thus, limited data exist on whether change in later life differs by race.^7,23,26,32^ Physical performance relies on multiple physiological systems^1^ and may be influenced over time by these systems and socioeconomic influences, yet studies have not simultaneously assessed these factors and associations with physical performance declines by race. Applying a comprehensive approach is key to understanding racial disparities in physical performance to inform public screening and timely interventions to promote healthy aging.

Examining racial disparities, however, requires an understanding that race is a complex, multifaceted social construct representing various factors, including social, cultural, health-related, and geographical influences.^33^ Furthermore, as racial health differences exhibit geographical variation, consideration of geographical influences on racial disparities in health is needed. For example, self-reported mobility disability is higher in the southern US among both Black and White residents compared with other states,^28,34,35^ with similar estimates among Black and White residents within southern states.^34^ This finding suggests that regional factors may be associated with function and mobility disability and potentially explain differences across racially defined groups.^34^ However, longitudinal studies with objective functional measures of physical performance in later life among underrepresented groups of people and associated risk factors by race and region are sparse.

The present study sought to examine lower-extremity physical performance changes over time by race and by race and region among older Black adults and older White adults from 4 communities in the US, while accounting for factors that are commonly suggested to be associated with performance differences.

## Methods

### Study Population

The Atherosclerosis Risk in Communities (ARIC) Study is a prospective study that recruited 15 792 adults, aged 45 to 64 years, between 1987 and 1989 (visit 1) from 4 US communities (Jackson, Mississippi; suburbs of Minneapolis, Minnesota; Forsyth County, North Carolina; and Washington County, Maryland).^36,37^ Participants were followed up via 9 cohort-wide in-person examinations, with the last examination completed in 2022. Physical performance was first assessed at the fifth in-person examination (visit 5, 2011-2013; hereafter, *baseline*), which 6538 participants attended; this assessment was repeated at the sixth (2016-2017), seventh (2018-2019), and ninth (2021-2022) examinations.^13^ Of the 6538 visit 5 participants, 5666 met eligibility criteria for analysis (details in the eMethods in Supplement 1). This study was approved by the institutional review boards at University of Mississippi Medical Center, Johns Hopkins Bloomberg School of Public Health, University of North Carolina at Chapel Hill, and University of Minnesota. All participants provided written informed consent. The study followed the Strengthening the Reporting of Observational Studies in Epidemiology (STROBE) reporting guideline for cohort studies.

### Physical Function Performance Measures

Physical performance was ascertained using the Short Physical Performance Battery (SPPB), with standardized protocols across ARIC sites.^2,3,13^ The SPPB included 3 components as previously described: (1) standing balance in 3 positions: feet parallel (side by side, offered if participants were unable to hold semitandem), semitandem, and tandem positions for up to 10 seconds each; (2) 5 timed chair stands without using arms; and (3) usual-pace gait speed assessed twice over a 4-m course, allowing walking aids for safety, if needed (the faster time from 2 trials was used to calculate gait speed [meters per second]).^3^ Balance, chair stand, and gait speed tasks were each scored on a range from 0 to 4 points (with higher scores indicating better performance) based on established thresholds; those who were unsafe or unable to complete a task were assigned a score of 0.^3^ We examined SPPB task scores, a composite SPPB score (the sum of the 3 component scores; range, 0-12), and, separately, continuous gait speed (meters per second) as physical performance measures.^2,3^ A composite SPPB score difference of 0.5 is considered a small change, and a score difference of 1.0 is considered a substantial clinically meaningful change.^38^ Gait speed differences of 0.04 to 0.05 meters per second are small and clinically meaningful, with a 0.1-meter-per-second slower gait speed considered a substantial clinically meaningful difference, associated with a 12% higher mortality.^1,38^

### Covariates

We included the following covariates that have been linked with both race and functional measures: (1) demographic factors: age and sex; (2) cardiovascular factors: cigarette smoking, alcohol use, obesity, diabetes, hypertension, heart failure, coronary heart disease, and stroke^10,13,39^; (3) socioeconomic status: educational level, family income, number of dependents, and census tract–level area deprivation index^12,17,19,40^; and (4) cognition: global cognitive scores and dementia status,^11,15^ both of which were statistically supported when included in the same model. Cognitive factors have been associated with decline in performance-based physical function measures.^15,41,42^ Participants’ race (Black or African American or White), sex, and educational level were self-reported at visit 1. All other covariates were obtained at visit 5 (eMethods in Supplement 1).

### Statistical Analysis

Statistical analysis took place from January through May 2025. We used multivariable generalized estimating equations to compare population-mean physical performance between Black participants and White participants using log-links and negative binomial distributions for the composite SPPB and subcomponents and using a log-link and gamma distribution for continuous gait speed. Race, time since visit 5, and an interaction term between race and time were used to examine race-associated trajectories of physical performance. We specified exchangeable correlation structures with Huber-White robust standard errors. Marginal standardization^43^ estimated absolute and relative differences in baseline scores, and separately in 10th-year outcome scores and in the 10-year rate of declines. To examine factors potentially explaining differences by race, we ran 4 series of regression models adjusting for age and sex (model 1), model 1 covariates plus cardiovascular factors (model 2), model 2 covariates plus socioeconomic factors (model 3), and model 3 covariates plus cognitive factors (model 4). To examine SPPB components potentially associated with differences by race in SPPB composite scores, analyses were repeated using each component as the outcome.

To examine hypothesized regional associations potentially accounting for race differences in physical performance decline, we created a 7-level categorical race-region variable (Black-Mississippi, Black-Maryland, Black-Minnesota, Black–North Carolina, White-Maryland, White-Minnesota, and White–North Carolina) and estimated differences in composite SPPB score 10-year decline associated with race and region, with White–North Carolina as the reference (due to having a reasonable number of Black participants and White participants), using model 4. To examine potential bias associated with loss to follow-up, we conducted sensitivity analyses using inverse probability of attrition weights (IPAW) under the missingness-at-random assumption and then joint shared parameter models (SPMs) under the extended missingness-at-random assumption.^44^ Analyses used STATA/SE, version 18.5 (StataCorp LLC). Statistical significance was defined as a 2-sided *P* < .05.

## Results

Among 5666 visit 5 participants (mean [SD] age, 75.4 [5.1] years; 3258 [58%] women and 2408 men [42%]), 1233 (22%) reported their race as Black and 4433 (78%) reported their race as White (Table 1). Compared with White participants, Black participants were younger, had a higher proportion of women, and had greater prevalence of obesity, diabetes, hypertension, heart failure, stroke, and cognitive impairment, but were less likely to have coronary heart disease or consume alcohol. Black participants had lower income and lived in neighborhoods with higher socioeconomic deprivation. Those attending all 3 follow-up visits were younger, with better baseline SPPB scores (eTables 1 and 2 in Supplement 1; median follow-up, 4.8 years, and maximum follow-up, 10.6 years) than those attending fewer than 3 follow-up visits.

**Table 1.** Distribution of Select Characteristics of Study Participants, Overall and by Race, at Visit 5 (2011-2013) of the ARIC Study

Visit 5 characteristics^a^	All participants (N = 5666)	Black participants (n = 1233)	White participants (n = 4433)	*P* value
Demographic characteristics				
Age, mean (SD), y	75.4 (5.1)	74.4 (4.9)	75.7 (5.1)	<.001
Sex, No. (%)				
Male	2408 (42)	426 (35)	1982 (45)	<.001
Female	3258 (58)	797 (65)	2451 (55)	
Cardiovascular factors, No. (%)				
Obesity	1955 (35)	562 (46)	1393 (31)	<.001
Diabetes	1566 (28)	467 (38)	1099 (25)	<.001
Hypertension	4167 (74)	1069 (87)	3098 (71)	<.001
Current smoker	321 (6)	81 (7)	240 (5)	.13
Current drinking	2818 (50)	258 (21)	2560 (58)	<.001
Heart failure	703 (12)	233 (19)	470 (11)	<.001
Coronary heart disease	869 (16)	124 (10)	745 (17)	<.001
Stroke	207 (4)	62 (5)	145 (3)	<.01
Socioeconomic status				
Any college education, No. (%)	2523 (45)	531 (43)	1992 (45)	.24
Median income, mean (SD), $	46 580.63 (28 014.72)	33 446.79 (27 670.38)	50 225.53 (27 003.01)	<.001
Dependents >1, No. (%)	3935 (69)	666 (54)	3269 (74)	<.001
ADI national rank, mean (SD)	50.88 (25.24)	82.61 (19.93)	42.04 (18.57)	<.001
Cognition				
Global cognition factor score, mean (SD)	0.09 (0.90)	−0.69 (0.88)	0.30 (0.78)	<.001
Dementia, No. (%)	193 (3)	61 (5)	132 (3)	<.01
Physical performance, mean (SD)				
Composite SPPB score (range, 0-12)	9.51 (2.40)	8.50 (2.80)	9.79 (2.19)	<.001
SPPB balance score (range, 0-4)	3.52 (1.01)	3.25 (1.24)	3.59 (0.93)	<.001
SPPB chair stands score (range, 0-4)	2.43 (1.27)	1.97 (1.30)	2.55 (1.23)	<.001
SPPB gait score (range, 0-4)	3.56 (0.75)	3.27 (0.92)	3.65 (0.67)	<.001
Gait speed, m/s	0.94 (0.22)	0.84 (0.22)	0.96 (0.22)	<.001

Abbreviations: ADI, Area Deprivation Index; ARIC, Atherosclerosis Risk in Communities; SPPB, Short Physical Performance Battery.

^a^ Missing values: diabetes (White, n = 42; Black, n = 19); hypertension (White, n = 48; Black, n = 6); current smokers (White, n = 44; Black, n = 7); current drinkers (White, n = 47; Black, n = 8); coronary heart disease (White, n = 79; Black, n = 15); stroke (White, n = 7; Black, n = 3); educational level (White, n = 7; Black, n = 2); income (White, n = 336; Black, n = 96); dependents (White, n = 90; Black, n = 19); ADI (White, n = 12; Black, n = 0); and global cognition factor score (White, n = 30; Black, n = 1).

### Between-Race Differences in Physical Performance Measures

**Figure 1.**
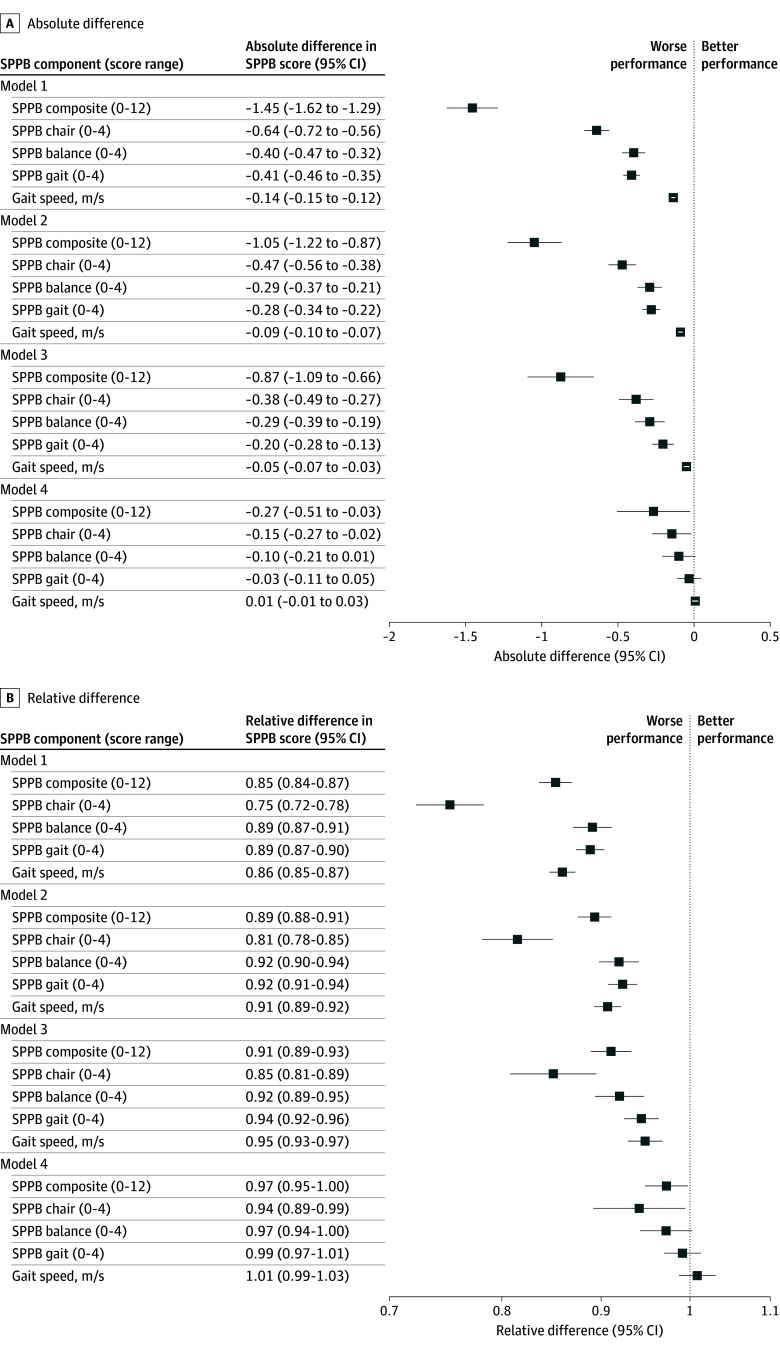
Forest Plot Showing Absolute and Relative Cross-Sectional Differences in Visit 5 (2011-2013) Composite Short Physical Performance Battery (SPPB) Score Between Black and White Participants in the Atherosclerosis Risk in Communities Study Model 1: adjusted for age and sex. Model 2: adjusted for model 1 covariates and cardiovascular factors (obesity, diabetes, hypertension, cigarette smoking, alcohol drinking, heart failure, coronary heart disease, and stroke). Model 3: adjusted for model 2 covariates and socioeconomic status (educational level, median income, number of dependents, and the national Area Deprivation Index). Model 4: adjusted for model 3 covariates and cognitive factors (global cognitive factor score and dementia).

**Figure 2.**
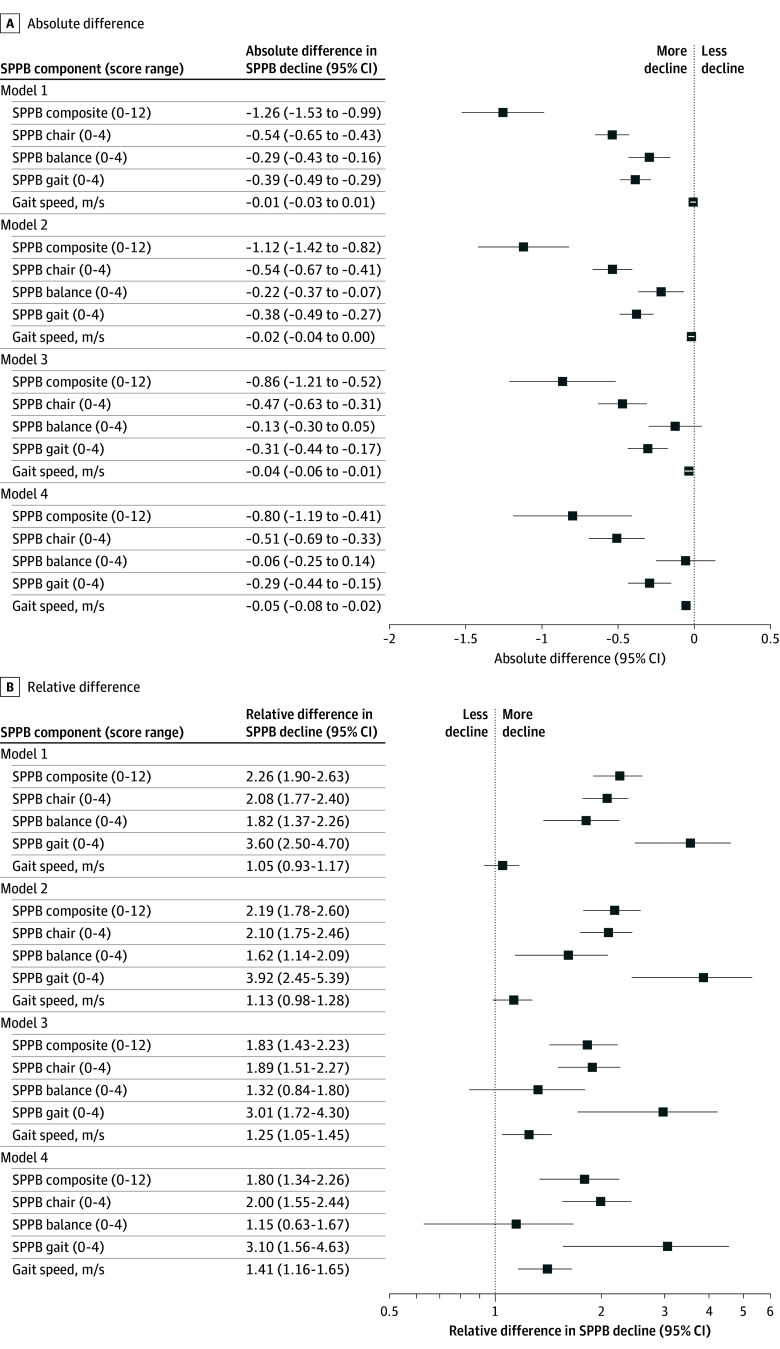
Forest Plot Showing Absolute and Relative Differences in the 10-Year Composite Short Physical Performance Battery (SPPB) Declines Between Black Participants and White Participants in the Atherosclerosis Risk in Communities Study Model 1: adjusted for age and sex. Model 2: adjusted for model 1 covariates and cardiovascular factors (obesity, diabetes, hypertension, cigarette smoking, alcohol drinking, heart failure, coronary heart disease, and stroke). Model 3: adjusted for model 2 covariates and socioeconomic status (educational level, median income, number of dependents, and the national Area Deprivation Index). Model 4: adjusted for model 3 covariates and cognitive factors (global cognitive factor score and dementia).

Baseline SPPB scores were worse across all measures for Black participants compared with White participants (Table 1; eFigure 1 in Supplement 1). The mean (SD) composite SPPB score was 8.50 (2.80) for Black participants and 9.79 (2.19) for White participants, and the mean (SD) gait speed was 0.84 (0.22) meters per second for Black participants vs 0.96 (0.22) meters per second for White participants. Both the composite SPPB score and gait speed exceeded thresholds for substantial clinically meaningful differences.^1,38^ Although these baseline racial differences were attenuated with successive adjustments for sociodemographic, cardiovascular, socioeconomic, and cognitive factors, racial differences in the composite SPPB score and chair stand task (Figure 1; Table 2) remained statistically different although not clinically meaningful. Black participants experienced faster declines than White participants for all measures but the balance task (model 4) (Table 2; Figure 2). The 10-year composite SPPB decline was greater for Black participants (relative difference, 1.80 [95% CI, 1.34-2.26], with a clinically meaningful absolute difference of −0.80 points [95% CI, −1.19 to −0.41 points]).^1^ Likewise, the mean 10-year gait speed decline was greater (relative difference, 1.41 [95% CI, 1.16-1.65]) for Black participants compared with White participants, with an absolute difference of −0.05 meters per second (95% CI, −0.08 to −0.02 meters per second). The results were similar in sensitivity analyses accounting for cohort attrition using IPAW (eFigure 2 in Supplement 1) and SPM models. Differences in SPPB measures between Black participants and White participants at the 10-year follow-up widened relative to baseline differences for all measures except the balance task (Table 2).

**Table 2.** Associations of Race With Physical Performance at Index Examination (Visit 5), at 10-Year Follow-Up, and With the 10-Year Decline in the ARIC Study^a^

Physical performance measures	Mean score		Difference (95% CI)		*P* value
	Black participants	White participants	Absolute (Black-White)	Relative (Black compared to White [reference])	
**At visit 5**					
SPPB score (range, 0-12)	9.39	9.66	−0.27 (−0.51 to −0.03)	0.97 (0.95 to 1.00)	.03
SPPB chair stands score (range, 0-4)	2.34	2.49	−0.15 (−0.27 to −0.02)	0.94 (0.89 to 0.99)	.03
SPPB balance score (range, 0-4)	3.47	3.57	−0.10 (−0.21 to 0.01)	0.97 (0.94 to 1.00)	.07
SPPB gait score (range, 0-4)	3.57	3.60	−0.03 (−0.11 to 0.05)	0.99 (0.97 to 1.01)	.43
Gait speed (m/s)	0.95	0.94	0.01 (−0.01 to 0.03)	1.01 (0.99 to 1.03)	.43
**At 10-year follow-up**					
SPPB score (range, 0-12)	7.59	8.67	−1.07 (−1.47 to −0.67)	0.88 (0.83 to 0.92)	<.001
SPPB chair stands score (range, 0-4)	1.33	2.01	−0.68 (−0.85 to −0.51)	0.66 (0.59 to 0.75)	<.001
SPPB balance score (range, 0-4)	3.03	3.20	−0.16 (−0.35 to 0.02)	0.95 (0.89 to 1.01)	.10
SPPB gait score (range, 0-4)	3.14	3.47	−0.33 (−0.47 to −0.19)	0.91 (0.87 to 0.95)	<.001
Gait speed (m/s)	0.76	0.81	−0.05 (−0.07 to −0.02)	0.94 (0.91 to 0.98)	<.01
**10-y Declines**					
Composite SPPB score (range, 0-12)	−1.80	−1.00	−0.80 (−1.19 to −0.41)	1.80 (1.34 to 2.26)	<.001
SPPB chair stands score (range, 0-4)	−1.02	−0.51	−0.51 (−0.69 to −0.33)	2.00 (1.55 to 2.44)	<.001
SPPB balance score (range, 0-4)	−0.44	−0.38	−0.06 (−0.25 to 0.14)	1.15 (0.63 to 1.67)	.57
SPPB gait score (range, 0-4)	−0.43	−0.14	−0.29 (−0.44 to −0.15)	3.10 (1.56 to 4.63)	<.001
Gait speed, m/s	−0.19	−0.13	−0.05 (−0.08 to −0.02)	1.41 (1.16 to 1.65)	<.001

Abbreviations: ARIC, Atherosclerosis Risk in Communities; SPPB, Short Physical Performance Battery.

^a^ Models were adjusted for age, sex, obesity, diabetes, hypertension, smoking status, heart failure, coronary heart disease, stroke, educational level, median income, number of dependents, Area Deprivation Index national rank, global cognition factor score, and dementia.

### Race-Region Differences in Physical Performance Decline

**Figure 3.**
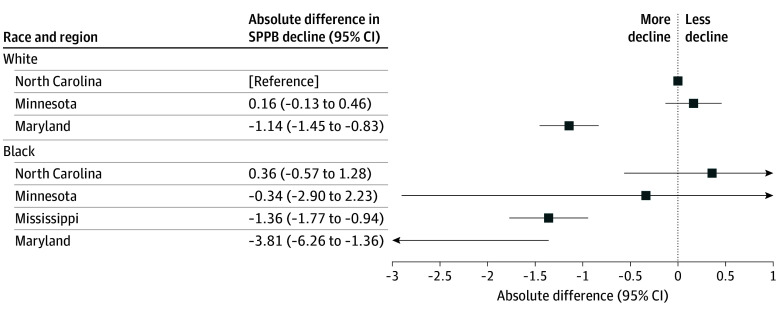
Forest Plot Showing Comparisons of the 10-Year Decline in the Composite Short Physical Performance Battery (SPPB) Score by Race and Region in the Atherosclerosis Risk in Communities Study The model was adjusted for age, sex, obesity, diabetes, hypertension, smoking status, heart failure, coronary heart disease, stroke, educational level, median income, number of dependents, the national rank Area Deprivation Index, global cognition factor score, and dementia. The figure shows absolute decline differences from White North Carolina participants (reference) with 95% CIs by race and region.

Although the analysis of race-region differences in SPPB decline was limited by the small number of Black participants in Maryland and Minnesota (eTable 3 and eFigure 3 in Supplement 1), we observed marked within-race, between-region differences in the composite SPPB decline (model 4) (Figure 3). Compared with White North Carolina participants, White Maryland participants had faster and clinically meaningful SPPB declines (absolute 10-year decline, −1.14 points [95% CI, −1.45 to −0.83 points]) (eTable 4 in Supplement 1). Among Black participants across study sites, Black participants in Mississippi and Maryland experienced greater absolute declines than those in North Carolina (Mississippi, −1.72 points [95% CI, −2.65 to −0.78 points]; and Maryland, −4.17 points [95% CI, −6.77 to −1.57 points]). The between-race difference was not supported within North Carolina or Minnesota, although there were a small number of Black participants in Minnesota (n = 9). Analyses incorporating IPAW showed similar results (eFigure 4 in Supplement 1).

## Discussion

In this cohort study of community-dwelling older adults, Black participants performed significantly worse than White participants across all physical performance measures, with substantial and clinically meaningful^38^ lower composite SPPB scores and slower gait speeds; cross-sectional differences were largely explained by demographic, cardiovascular, socioeconomic, and cognitive factors. However, even after adjusting for these factors, Black participants experienced steeper 10-year physical performance declines and had worse performance at the end of the follow-up period. Within-race, between-region variations, with greater composite SPPB declines among White Maryland participants compared with White North Carolina participants and among Black Mississippi participants compared with Black North Carolina participants, suggest that unmeasured factors related to geographic regions, apart from those examined in this cohort, may account for aging-related racial differences in physical performance declines.

Our results extend existing literature suggesting that cardiovascular factors, socioeconomic status, and cognition^7,21,25,26^ may account for racial differences in short-term, longitudinal decline to some extent by reporting novel findings suggesting that unmeasured region-specific factors may explain racial differences in long-term lower-extremity performance declines. Understanding this gap could be key to preserving physical performance abilities with aging regardless of race. Among studies with objective, longitudinal physical performance measures, 1 study^7^ reported a steeper 6-year decline in SPPB among Black participants compared with White participants, but the study did not account for comorbidities or cognition; the value of performance-based measures was evident, as racial differences in incident self-reported disability were not supported. Another study reported slower gait speed and steeper declines among Black participants compared with White participants, but declines were attenuated among those older than 65 years compared with those aged 53 to 64 years among Black participants, adjusting for demographics, body mass index, and income.^45^ Our study, in contrast, included adults aged 65 years or older and adjusted for extended covariates. The differences in decline by race among ARIC participants could also be due to ARIC examining a short, usual-pace walk while the other study assessed a faster gait speed over a long-distance test designed to assess endurance in older adults. Another large cohort^26^ did not find differences in gait speed decline (defined using a cutoff) between Black participants and White participants. However, the study recruited individuals without self-reported mobility limitations from a narrow age range (70-79 years). The persisting race differences in SPPB and gait speed decline in ARIC could be related to the longer follow-up, use of a continuous rather than a dichotomous gait speed measure, broader age spectrum (65-84 years), and poorer mobility at the index examination. Some studies using self-reported measures of function have reported no differential decline between Black and White individuals after adjusting for varying covariates,^23,32,46^ which likely suggests that performance-based measures are more sensitive measures of decline than self-reported measures.^47^

Cross-sectional studies have also suggested that cardiovascular, socioeconomic, and/or cognitive factors may explain racial disparities in objective^20,21,22,25^ or subjective^24,27,28,29,30,31^ functional measures, but they have been inconsistent with varying potential confounders considered.^24,27,30^ Few studies accounted for cognition along with cardiovascular and socioeconomic factors, and those with objective physical function measures generally showed persisting poorer performance among Black participants.^7,21^ Racial differences in physical performance increased over the follow-up in ARIC along with accelerated functional declines among Black participants, which could have several explanations. One theory posits that cumulative life-course advantages and disadvantages may have long-lasting influences on health trajectories and contribute to disparities widening over time.^32,48^ Time-varying elements, including higher rates of cardiovascular factors^49^; cognitive decline^10^; adverse socioeconomic factors associated with income, health care access, psychosocial stress, and nutritional factors^32,50,51^; or differential burdens in other health outcomes, including musculoskeletal diseases, may also partly explain the residual differences.^26,52^ Overall, attenuation in between-race differences in physical performance after adjusting for cardiovascular, socioeconomic, and cognitive factors observed in this and prior studies^7,21,25,26^ underscore that these factors, individually or in clusters, may identify individuals at risk of functional decline. Because many of these factors are modifiable, they could serve as intervention targets for the maintenance of physical functioning and reducing racial disparities in physical functioning.

Furthermore, our analyses support other work^28,35^ suggesting regional influences are vital to consider when examining racial differences in physical performance. Rates of cardiovascular risk factors and disease,^10^ cognitive impairment,^53^ and socioeconomic factors differ across regions, with higher rates generally observed in southern states and potentially explaining some regional differences. Although others have observed regional differences,^28,35,54,55,56^ we extend this prior work, which was limited by self-reported functional status and limited or no assessments of comorbidities, cognition, or longitudinal measures.^54^ Black and White residents from the southern US have a higher prevalence of self-reported disability than those in northern states, although differences varied by region and study.^28,34,35^ For example, in the 2022 Behavioral Risk Factor Surveillance System survey,^34^ although age-adjusted self-reported mobility disability was higher among Black residents than White residents in Minnesota, the mobility disability prevalence among White Mississippi residents was higher than Black Minnesota residents and double that of White Minnesota residents; mobility disability prevalence was similar by race in Mississippi. Higher mobility disability in southern-born Black individuals and White individuals persisted despite migration to other regions in 1 study,^35^ but only among White participants in another study.^28^ These and our results suggest that regional factors may have long-lasting but potentially modifiable associations with aging-related functional outcomes.

### Strengths and Limitations

Our study has some strengths. Major strengths include the large number of participants from 4 communities; objective, longitudinal measures of physical performance over 10 years; detailed cardiovascular phenotyping from midlife; comprehensive cognitive assessments; and socioeconomic measures beyond educational level and income, including area deprivation. Our study also has some limitations. Although this study is one of the few to elucidate physical performance differences across race and region, regions were limited to 4 US communities. However, our findings may be more broadly generalizable. ARIC field centers recruited from beyond the immediate community area. Furthermore, studies of stroke risk factors in the REGARDS (Reasons for Geographic and Racial Differences in Stroke) study found similar prevalence rates within states and even across neighboring states, with regional differences being similar or larger than racial differences, providing some support that the findings may not be exclusive to the communities examined.^57^ Our analyses were limited by small numbers of Black participants at 2 sites, and White participants were not recruited at the Mississippi site. Nonetheless, these findings can inform future studies of geographical differences in mobility and function. Validated clinically meaningful changes in SPPB have not been defined over 10 years of follow-up; however, our findings suggest the 0.80-point steeper decline among Black participants is meaningful when compared with the age estimate. A 10-year SPPB decline of 0.80 points in this study was comparable to having aged 20 years over a 10-year period. Our analysis included only Black and White adults, and future studies should include other racial and ethnic groups. We considered only late-life exposures with late-life outcomes and lacked detailed information on concurrent arthritis and psychosocial stressors. However, this is comparable with most aging-related longitudinal studies^26,45^; thus, comparisons across studies may be more alike than not. The analysis did not consider disease severity, which could explain residual differences, and ARIC lacked data on factors potentially associated with regional differences, such as environmental exposures, migration patterns (although almost all individuals remain at the same field center), social and contextual factors, and regional public health policies, which could have provided insights into potential contributors to the observed regional differences. Attrition, particularly if different across race centers, could bias results. However, we anticipate this would lead to conservative estimates, and results from sensitivity analyses that used IPAW and SPM were similar to the primary findings.

## Conclusions

In this cohort study of older adults, we observed pronounced steeper declines in performance-based physical function measures among Black adults compared with White adults that were not explained by well-phenotyped cardiovascular, cognitive, and sociodemographic factors; within-race differences across sites suggested that racial differences may be related to unmeasured regional exposures. Region-specific risk factors may drive geographical differences in functional status and critical periods when interventions could mitigate adverse functional outcomes; these are important gaps in knowledge that warrant investigation.

## Notes

Supplement 1.
eMethods.

eReferences.
**eTable 1.** Distribution of Select Characteristics of Study Participants at Visit 5 (2011-2013), Overall and by the Number of Follow-Up Attendances **eTable 2.** Distribution of Select Characteristics of Study Participants at Visit 5 (2011-2013), by Race and by the Number of Follow-Up Attendances **eTable 3.** Distribution of Select Characteristics of Study Participants at Visit 5 (2011-2013), by Race and Region **eTable 4.** Pairwise Absolute and Relative Differences in 10-Year Composite SPPB Declines Between Regions Within Race **eFigure 1.** Trajectories of the Composite SPPB Score Over Time by Race in the ARIC Study **eFigure 2.** Absolute and Relative Differences in the 10-Year Composite SPPB Declines Between Black and White Participants in the ARIC Study Applying Inverse Probability Weights for Attrition **eFigure 3.** Comparisons of the Composite SPPB Score Differences at Visit 5 (Baseline) by Race and Region in the ARIC Study **eFigure 4.** Comparisons of the 10-Year Decline in the Composite SPPB Score by Race and Region in the ARIC Study Incorporating Inverse Probability Weights for Attrition

Supplement 2.
Data Sharing Statement

## References

[1] Studenski S, Perera S, Patel K, . Gait speed and survival in older adults. JAMA. 2011;305(1):50-58. doi:10.1001/jama.2010.1923 21205966 PMC3080184

[2] Guralnik JM, Ferrucci L, Simonsick EM, Salive ME, Wallace RB. Lower-extremity function in persons over the age of 70 years as a predictor of subsequent disability. N Engl J Med. 1995;332(9):556-561. doi:10.1056/NEJM199503023320902 7838189 PMC9828188

[3] Guralnik JM, Simonsick EM, Ferrucci L, . A short physical performance battery assessing lower extremity function: association with self-reported disability and prediction of mortality and nursing home admission. J Gerontol. 1994;49(2):M85-M94. doi:10.1093/geronj/49.2.M85 8126356

[4] Fusco O, Ferrini A, Santoro M, Lo Monaco MR, Gambassi G, Cesari M. Physical function and perceived quality of life in older persons. Aging Clin Exp Res. 2012;24(1):68-73. doi:10.1007/BF03325356 22643307

[5] Jacob ME, Marron MM, Boudreau RM, Odden MC, Arnold AM, Newman AB. Age, race, and gender factors in incident disability. J Gerontol A Biol Sci Med Sci. 2018;73(2):194-197. doi:10.1093/gerona/glx194 29045556 PMC5861898

[6] Haas SA, Krueger PM, Rohlfsen L. Race/ethnic and nativity disparities in later life physical performance: the role of health and socioeconomic status over the life course. J Gerontol B Psychol Sci Soc Sci. 2012;67(2):238-248. doi:10.1093/geronb/gbr155 22391749 PMC3410696

[7] Mendes de Leon CF, Barnes LL, Bienias JL, Skarupski KA, Evans DA. Racial disparities in disability: recent evidence from self-reported and performance-based disability measures in a population-based study of older adults. J Gerontol B Psychol Sci Soc Sci. 2005;60(5):S263-S271. doi:10.1093/geronb/60.5.S263 16131627

[8] Dong L, Freedman VA, Sánchez BN, Mendes de Leon CF. Racial and ethnic differences in disability transitions among older adults in the United States. J Gerontol A Biol Sci Med Sci. 2019;74(3):406-411. doi:10.1093/gerona/gly052 29562316 PMC6376147

[9] Minhas AMK, Talha KM, Abramov D, . Racial and ethnic disparities in cardiovascular disease—analysis across major US national databases. J Natl Med Assoc. 2024;116(3):258-270. doi:10.1016/j.jnma.2024.01.022 38342731 PMC13012869

[10] Martin SS, Aday AW, Almarzooq ZI, ; American Heart Association Council on Epidemiology and Prevention Statistics Committee and Stroke Statistics Subcommittee. 2024 Heart disease and stroke statistics: a report of US and global data from the American Heart Association. Circulation. 2024;149(8):e347-e913. doi:10.1161/CIR.0000000000001209 38264914 PMC12146881

[11] Power MC, Bennett EE, Turner RW, . Trends in relative incidence and prevalence of dementia across Non-Hispanic Black and White individuals in the United States, 2000-2016. JAMA Neurol. 2021;78(3):275-284. doi:10.1001/jamaneurol.2020.4471 33252617 PMC7953306

[12] Baker RS, Brady D, Parolin Z, Williams DT. The enduring significance of ethno-racial inequalities in poverty in the U.S., 1993–2017. Popul Res Policy Rev. 2022;41(3):1049-1083. doi:10.1007/s11113-021-09679-y

[13] Windham BG, Harrison KL, Lirette ST, . Relationship between midlife cardiovascular health and late-life physical performance: the ARIC Study. J Am Geriatr Soc. 2017;65(5):1012-1018. doi:10.1111/jgs.14732 28165626 PMC5435564

[14] Windham BG, Griswold ME, Wang W, . The importance of mid-to-late-life body mass index trajectories on late-life gait speed. J Gerontol A Biol Sci Med Sci. 2017;72(8):1130-1136. doi:10.1093/gerona/glw200 27811156 PMC5861851

[15] Buchman AS, Boyle PA, Leurgans SE, Barnes LL, Bennett DA. Cognitive function is associated with the development of mobility impairments in community-dwelling elders. Am J Geriatr Psychiatry. 2011;19(6):571-580. doi:10.1097/JGP.0b013e3181ef7a2e 21606900 PMC3101472

[16] Dhamoon MS, Dong C, Elkind MS, Sacco RL. Ideal cardiovascular health predicts functional status independently of vascular events: the Northern Manhattan Study. J Am Heart Assoc. 2015;4(2):e001322. doi:10.1161/JAHA.114.001322 25677566 PMC4345864

[17] Coppin AK, Ferrucci L, Lauretani F, . Low socioeconomic status and disability in old age: evidence from the InChianti Study for the mediating role of physiological impairments. J Gerontol A Biol Sci Med Sci. 2006;61(1):86-91. doi:10.1093/gerona/61.1.86 16456198

[18] Zimmer Z, House JS. Education, income, and functional limitation transitions among American adults: contrasting onset and progression. Int J Epidemiol. 2003;32(6):1089-1097. doi:10.1093/ije/dyg254 14681281

[19] Zaninotto P, Sacker A, Head J. Relationship between wealth and age trajectories of walking speed among older adults: evidence from the English Longitudinal Study of Ageing. J Gerontol A Biol Sci Med Sci. 2013;68(12):1525-1531. doi:10.1093/gerona/glt058 23682157 PMC3814237

[20] Blanco I, Verghese J, Lipton RB, Putterman C, Derby CA. Racial differences in gait velocity in an urban elderly cohort. J Am Geriatr Soc. 2012;60(5):922-926. doi:10.1111/j.1532-5415.2012.03927.x 22587854 PMC3354735

[21] Clay OJ, Thorpe RJ Jr, Wilkinson LL, . An examination of lower extremity function and its correlates in older African American and White men. Ethn Dis. 2015;25(3):271-278. doi:10.18865/ed.25.3.271 26673095 PMC4671416

[22] Kirkness CS, Ren J. Race differences: use of walking speed to identify community-dwelling women at risk for poor health outcomes—Osteoarthritis Initiative Study. Phys Ther. 2015;95(7):955-965. doi:10.2522/ptj.20140028 25655879 PMC4498144

[23] Jacobs JC, Bowling CB, Brown T, . Racial inequality in functional trajectories between Black and White U.S. veterans. J Am Geriatr Soc. 2023;71(4):1081-1092. doi:10.1111/jgs.18169 36519710 PMC10089950

[24] Louie GH, Ward MM. Socioeconomic and ethnic differences in disease burden and disparities in physical function in older adults. Am J Public Health. 2011;101(7):1322-1329. doi:10.2105/AJPH.2010.199455 21164082 PMC3110229

[25] Sternfeld B, Colvin A, Stewart A, . Understanding racial/ethnic disparities in physical performance in midlife women: findings from SWAN (Study of Women’s Health Across the Nation). J Gerontol B Psychol Sci Soc Sci. 2020;75(9):1961-1971. doi:10.1093/geronb/gbz103 31412129 PMC7566973

[26] Thorpe RJ Jr, Koster A, Kritchevsky SB, ; Health, Aging, and Body Composition Study. Race, socioeconomic resources, and late-life mobility and decline: findings from the Health, Aging, and Body Composition Study. J Gerontol A Biol Sci Med Sci. 2011;66(10):1114-1123. doi:10.1093/gerona/glr102 21743093 PMC3172564

[27] Wright KD, Pepper GA, Caserta M, . Factors that influence physical function and emotional well-being among Medicare-Medicaid enrollees. Geriatr Nurs. 2015;36(2)(suppl):S16-S20. doi:10.1016/j.gerinurse.2015.02.022 25784082 PMC4393784

[28] Kington R, Carlisle D, McCaffrey D, Myers H, Allen W. Racial differences in functional status among elderly U.S. migrants from the South. Soc Sci Med. 1998;47(6):831-840. doi:10.1016/S0277-9536(98)00145-2 9690828

[29] Luck J, Govier D, Ðoàn LN, Mahakalanda S, Zhang W, Mendez-Luck C. Functional limitations and physical health in community-dwelling Medicare Advantage beneficiaries: variation by race and Hispanic subgroup. J Aging Health. 2022;34(9-10):1269-1280. doi:10.1177/08982643221113133 36175065

[30] Vásquez E, Germain CM, Tang F, Lohman MC, Fortuna KL, Batsis JA. The role of ethnic and racial disparities in mobility and physical function in older adults. J Appl Gerontol. 2020;39(5):502-508. doi:10.1177/0733464818780631 29909728

[31] Brenner AB, Clarke PJ. Understanding socioenvironmental contributors to racial and ethnic disparities in disability among older Americans. Res Aging. 2018;40(2):103-130. doi:10.1177/0164027516681165 27909061 PMC6500723

[32] Kelley-Moore JA, Ferraro KF. The Black/White disability gap: persistent inequality in later life? J Gerontol B Psychol Sci Soc Sci. 2004;59(1):S34-S43. doi:10.1093/geronb/59.1.S34 14722342

[33] National Academies of Sciences, Engineering, and Medicine. *Rethinking Race and Ethnicity in Biomedical Research*. The National Academies Press; 2025.

[34] Disability and Health Data System (DHDS). Centers for Disease Control and Prevention. Accessed December 29, 2024. https://dhds.cdc.gov

[35] Lin G. Regional assessment of elderly disability in the U.S. Soc Sci Med. 2000;50(7-8):1015-1024. doi:10.1016/S0277-9536(99)00351-2 10714923

[36] ARIC Investigators. The Atherosclerosis Risk in Communities (ARIC) Study: design and objectives: the ARIC investigators. Am J Epidemiol. 1989;129(4):687-702. doi:10.1093/oxfordjournals.aje.a115184 2646917

[37] Wright JD, Folsom AR, Coresh J, . The ARIC (Atherosclerosis Risk In Communities) Study: JACC Focus Seminar 3/8. J Am Coll Cardiol. 2021;77(23):2939-2959. doi:10.1016/j.jacc.2021.04.035 34112321 PMC8667593

[38] Perera S, Mody SH, Woodman RC, Studenski SA. Meaningful change and responsiveness in common physical performance measures in older adults. J Am Geriatr Soc. 2006;54(5):743-749. doi:10.1111/j.1532-5415.2006.00701.x 16696738

[39] Skow LF, Sharrett AR, Gottesman RF, . Mid-life vascular risk and rate of physical function decline among older adults: the Atherosclerosis Risk in Communities (ARIC) Study. J Gerontol A Biol Sci Med Sci. 2024;79(2):glad210. doi:10.1093/gerona/glad210 37659100 PMC10809050

[40] Parker KG, Windham BG, Blackshear C, . Associations of mid-to-late-life inflammation with late-life mobility and the influences of chronic comorbidities, race, and social determinants of health: the Atherosclerosis Risk in Communities Study. J Am Geriatr Soc. 2024;72(8):2434-2445. doi:10.1111/jgs.18978 38863338 PMC11323257

[41] Tian Q, An Y, Resnick SM, Studenski S. The relative temporal sequence of decline in mobility and cognition among initially unimpaired older adults: results from the Baltimore Longitudinal Study of Aging. Age Ageing. 2017;46(3):445-451. doi:10.1093/ageing/afw185 27744302 PMC5860013

[42] Tian Q, Resnick SM, Davatzikos C, . A prospective study of focal brain atrophy, mobility and fitness. J Intern Med. 2019;286(1):88-100. doi:10.1111/joim.12894 30861232 PMC6586507

[43] Localio AR, Henegan JA, Chang S, . Standardization and prediction to control confounding: estimating risk differences and ratios for clinical interpretations and decision making. Ann Intern Med. 2025;178(6):829-835. doi:10.7326/ANNALS-25-00082 40194286

[44] Griswold ME, Talluri R, Zhu X, . Reflection on modern methods: shared-parameter models for longitudinal studies with missing data. Int J Epidemiol. 2021;50(4):1384-1393. doi:10.1093/ije/dyab086 34113988 PMC8407871

[45] Ip EH, Chen SH, Rejeski WJ, . Gradient and acceleration of decline in physical and cognitive functions in older adults: a disparity analysis. J Gerontol A Biol Sci Med Sci. 2022;77(8):1603-1611. doi:10.1093/gerona/glac109 35562076 PMC9373944

[46] Brown TH. Racial stratification, immigration, and health inequality: a life course-intersectional approach. Soc Forces. 2018;96(4):1507-1540. doi:10.1093/sf/soy013

[47] Simonsick EM, Newman AB, Nevitt MC, ; Health ABC Study Group. Measuring higher level physical function in well-functioning older adults: expanding familiar approaches in the Health ABC study. J Gerontol A Biol Sci Med Sci. 2001;56(10):M644-M649. doi:10.1093/gerona/56.10.M644 11584038

[48] Shuey KM, Willson AE. Cumulative disadvantage and Black-White disparities in life-course health trajectories. Res Aging. 2008;30(2):200-225. doi:10.1177/0164027507311151

[49] Mayeda ER, Haan MN, Neuhaus J, . Type 2 diabetes and cognitive decline over 14 years in middle-aged African Americans and Whites: the ARIC Brain MRI Study. Neuroepidemiology. 2014;43(3-4):220-227. doi:10.1159/000366506 25402639 PMC4370220

[50] Yearby R. Racial disparities in health status and access to healthcare: the continuation of inequality in the United States due to structural racism. Am J Econ Sociol. 2018;77(3-4):1113-1152. doi:10.1111/ajes.12230

[51] Chandra A, Skinner J. Geography and racial health disparities. *National Bureau of Economic Research Working Paper Series*. 2003; no. 9513. doi:10.3386/w9513

[52] Allen KD. Racial and ethnic disparities in osteoarthritis phenotypes. Curr Opin Rheumatol. 2010;22(5):528-532. doi:10.1097/BOR.0b013e32833b1b6f 20473172

[53] Dhana K, Beck T, Desai P, Wilson RS, Evans DA, Rajan KB. Prevalence of Alzheimer’s disease dementia in the 50 US states and 3142 counties: a population estimate using the 2020 bridged-race postcensal from the National Center for Health Statistics. Alzheimers Dement. 2023;19(10):4388-4395. doi:10.1002/alz.13081 37458371 PMC10593099

[54] Mendes de Leon CF, Beckett LA, Fillenbaum GG, . Black-white differences in risk of becoming disabled and recovering from disability in old age: a longitudinal analysis of two EPESE populations. Am J Epidemiol. 1997;145(6):488-497. doi:10.1093/oxfordjournals.aje.a009136 9063338

[55] Mendes de Leon CF, Fillenbaum GG, Williams CS, Brock DB, Beckett LA, Berkman LF. Functional disability among elderly Blacks and Whites in two diverse areas: the New Haven and North Carolina EPESE: Established Populations for the Epidemiologic Studies of the Elderly. Am J Public Health. 1995;85(7):994-998. doi:10.2105/AJPH.85.7.994 7604929 PMC1615553

[56] Vega WA, Sribney WM, Ayala SG. Regional disparities in ADL limitations among older Latinos, Blacks, and Whites in the United States. In: Vega WA, Angel JL, Gutiérrez Robledo LMF, Markides KS, eds. *Contextualizing Health and Aging in the Americas: Effects of Space, Time and Place*. Springer International Publishing; 2019:19-38.

[57] Howard G, Howard VJ. Twenty years of progress toward understanding the stroke belt. Stroke. 2020;51(3):742-750. doi:10.1161/STROKEAHA.119.024155 32078485

