# Supplementary material for: Physical Performance Changes Across Race and Region Among Black and White Older Adults
Source: JAMA Netw Open. 2026 Apr 30;9(4):e269937. doi: 10.1001/jamanetworkopen.2026.9937 (PMC13133694; doi:10.1001/jamanetworkopen.2026.9937)
Supplement: Supplement 1. — eMethods. eReferences. eTable 1. Distribution of Select Characteristics of Study Participants at Visit 5 (2011-2013), Overall and by the Number of Follow-Up Attendances eTable 2. Distribution of Select Characteristics of Study Participants at Visit 5 (2011-2013), by Race and by the Number of Follow-Up Attendances eTable 3. Distribution of Select Characteristics of Study Participants at Visit 5 (2011-2013), by Race and Region eTable 4. Pairwise Absolute and Relative Differences in 10-Year Composite SPPB Declines Between Regions Within Race eFigure 1. Trajectories of the Composite SPPB Score Over Time by Race in the ARIC Study eFigure 2. Absolute and Relative Differences in the 10-Year Composite SPPB Declines Between Black and White Participants in the ARIC Study Applying Inverse Probability Weights for Attrition eFigure 3. Comparisons of the Composite SPPB Score Differences at Visit 5 (Baseline) by Race and Region in the ARIC Study eFigure 4. Comparisons of the 10-Year Decline in the Composite SPPB Score by Race and Region in the ARIC Study Incorporating Inverse Probability Weights for Attrition [file jamanetwopen-e269937-s001.pdf]

Supplementary Online Content

Shrestha S, Windham W, Sylvester HC, et al. Physical performance changes across race and region among black and white older adults. *JAMA Netw Open.* 2026;9(4):e269937. doi:10.1001/jamanetworkopen.2026.9937

eMethods.
eReferences.
eTable 1. Distribution of Select Characteristics of Study Participants at Visit 5 (2011-2013), Overall and by the Number of Follow-Up Attendances
eTable 2. Distribution of Select Characteristics of Study Participants at Visit 5 (2011-2013), by Race and by the Number of Follow-Up Attendances
eTable 3. Distribution of Select Characteristics of Study Participants at Visit 5 (2011-2013), by Race and Region
eTable 4. Pairwise Absolute and Relative Differences in 10-Year Composite SPPB Declines Between Regions Within Race
eFigure 1. Trajectories of the Composite SPPB Score Over Time by Race in the ARIC Study
eFigure 2. Absolute and Relative Differences in the 10-Year Composite SPPB Declines Between Black and White Participants in the ARIC Study Applying Inverse Probability Weights for Attrition
eFigure 3. Comparisons of the Composite SPPB Score Differences at Visit 5 (Baseline) by Race and Region in the ARIC Study
eFigure 4. Comparisons of the 10-Year Decline in the Composite SPPB Score by Race and Region in the ARIC Study Incorporating Inverse Probability Weights for Attrition

This supplementary material has been provided by the authors to give readers additional information about their work.

© 2026 Shrestha S et al. *JAMA Network Open.*

eMethods.

Study Population

Of the 6,538 participants who attended visit 5, we excluded those who did not complete visit 5 physical function tests (n=789), self-reported race other than Black or White due to small numbers (n=13), and those having a body mass index (BMI) less than 18.5 kg/m^2 (n = 59) or missing BMI (n=15) at visit 5, resulting in a final analytic sample of 5,666. Of the 5,666 participants, physical performance was completed by 3,501 at visit 6, 3,173 at visit 7, and 1,863 at visit 9.

Covariates

Cigarette smoking and drinking status, total family income, and the number of people supported by the income were self-reported. Obesity was defined as a body mass index (BMI) ≥ 30 kg/m^2. Diabetes was defined as a fasting blood glucose of ≥ 126 mg/dL, a non-fasting blood glucose of ≥ 200 mg/dL, or anti-diabetic medication use. Hypertension was defined as a systolic blood pressure ≥ 140 mmHg, a diastolic blood pressure ≥ 90 mmHg, or the use of anti-hypertensive medications. Heart failure, stroke, and coronary heart disease were defined as a physician-adjudicated event identified through study surveillance and clinical review of medical records. The national area deprivation index (ADI), which provides standardized rankings (ranging from 0 to 100) of socioeconomic deprivation (derived using census tract data on employment, income, education, and housing quality) at the US census-tract level, was used as a measure of neighborhood-level socioeconomic disadvantage;[1,2] higher ADI score indicates greater deprivation. Global cognition factor scores were derived from ten neuropsychological tests (administered in-person) using a latent variable approach.[3] Dementia status was determined according to the Diagnostic and Statistical Manual of Mental Disorders, 5th Edition definition using an established protocol as previously described based on a neuropsychological test battery from in-person examinations, informant interviews, telephone interviews of participants and informants for participants who did not attend in-person exams, hospital discharge records, and review by an expert adjudication committee.[4]

© 2026 Shrestha S et al. *JAMA Network Open.*

eReferences.

1. Kind AJ, Jencks S, Brock J, et al. Neighborhood socioeconomic disadvantage and 30-day rehospitalization: a retrospective cohort study. *Ann Intern Med*. Dec 2 2014;161(11):765-74. doi:10.7326/M13-2946
2. Singh GK. Area deprivation and widening inequalities in US mortality, 1969-1998. *Am J Public Health*. Jul 2003;93(7):1137-43. doi:10.2105/ajph.93.7.1137
3. Gross AL, Power MC, Albert MS, et al. Application of Latent Variable Methods to the Study of Cognitive Decline When Tests Change over Time. *Epidemiology*. Nov 2015;26(6):878-87. doi:10.1097/EDE.0000000000000379
4. Knopman DS, Gottesman RF, Sharrett AR, et al. Mild Cognitive Impairment and Dementia Prevalence: The Atherosclerosis Risk in Communities Neurocognitive Study (ARIC-NCS). *Alzheimers Dement (Amst)*. 2016;2:1-11. doi:10.1016/j.dadm.2015.12.002

© 2026 Shrestha S et al. *JAMA Network Open.*

eTable 1. Distribution of Select Characteristics of Study Participants at Visit 5 (2011-2013), Overall and by the Number of Follow-Up Attendances

**Visit 5 Characteristics**	**Total attending Visit 5 (n = 5666)**	**No follow-up attended (n = 2186)**	**1 Follow-Up attended (n = 997)**	**2 Follow-Ups attended (n = 1337)**	**3 Follow-Ups attended (n = 1146)**
Demographics					
Age (years) [mean (SD)]	75.39 (5.07)	77.09 (5.39)	75.61 (4.85)	74.64 (4.56)	72.84 (3.74)
Male, n (%)	2408 (42%)	931 (43%)	417 (42%)	593 (44%)	467 (41%)
Female, n (%)	3258 (58%)	1255 (57%)	580 (58%)	744 (56%)	679 (59%)
Cardiovascular					
Obesity, n (%)	1955 (35%)	735 (34%)	354 (36%)	457 (34%)	409 (36%)
Diabetes, n (%)	1566 (28%)	702 (33%)	280 (29%)	320 (24%)	264 (23%)
Hypertension, n (%)	4167 (74%)	1686 (78%)	745 (75%)	939 (71%)	797 (70%)
Current Smokers, n (%)	321 (6%)	138 (6%)	59 (6%)	72 (5%)	52 (5%)
Current Drinking Status, n (%)	2818 (50%)	992 (46%)	495 (50%)	704 (53%)	627 (55%)
Heart Failure, n (%)	703 (12%)	387 (18%)	125 (13%)	119 (9%)	72 (6%)
Heart Disease, n (%)	869 (16%)	410 (19%)	166 (17%)	180 (14%)	113 (10%)
Stroke, n (%)	207 (4%)	107 (5%)	41 (4%)	43 (3%)	16 (1%)
Socioeconomic Status					
Any College Education, n (%)	2523 (45%)	866 (40%)	416 (42%)	654 (49%)	587 (51%)
Median Income ($) [mean (SD)]	46580.63 (28014.72)	41377.29 (26992.35)	46901.20 (27528.46)	49367.61 (28325.10)	52494.91 (28253.88)
Dependents > 1, n (%)	3935 (69%)	1447 (66%)	699 (70%)	966 (72%)	823 (72%)
ADI National Rank [mean (SD)]	50.88 (25.24)	51.19 (25.65)	51.13 (24.19)	51.54 (25.80)	49.30 (24.66)
Cognition					
Cognition Score [mean (SD)]	0.09 (0.90)	-0.24 (0.93)	0.09 (0.86)	0.29 (0.80)	0.48 (0.74)
Dementia, n (%)	193 (3%)	164 (8%)	20 (2%)	8 (1%)	1 (0%)
Physical Performance					
SPPB (0-12)	9.51 (2.40)	8.76 (2.76)	9.56 (2.29)	9.93 (2.01)	10.40 (1.58)
SPPB Balance (0-4)	3.52 (1.01)	3.25 (1.21)	3.58 (0.96)	3.66 (0.85)	3.81 (0.64)
SPPB Chair Stands (0-4)	2.43 (1.27)	2.16 (1.33)	2.41 (1.26)	2.58 (1.20)	2.78 (1.10)
SPPB Gait (0-4)	3.56 (0.75)	3.36 (0.88)	3.58 (0.73)	3.69 (0.62)	3.80 (0.47)
Gait speed (m/sec) [mean (SD)]	0.94 (0.22)	0.87 (0.23)	0.93 (0.21)	0.98 (0.22)	1.02 (0.21)

Abbreviations: ADI, Area Deprivation Index; SD, Standard Deviation; SPPB, Short Physical Performance Battery

Note: 'No follow-up attended' indicates that these participants attended Visit 5 exam (2011-2013) but did not attend any follow-up visits, and '3 Follow-Ups attended' indicates they attended all three follow-ups

eTable 2. Distribution of Select Characteristics of Study Participants at Visit 5 (2011-2013), by Race and by the Number of Follow-Up Attendances

	White participants					Black participants				
**Visit 5 Characteristics**	**Total attending Visit 5 (n=4433)**	**No Follow-Up attended (n=1712)**	**1 Follow-Up attended (n=794)**	**2 Follow-Ups attended (n=1036)**	**3 Follow-Ups attended (n=891)**	**Total attending Visit 5 (n=1233)**	**No Follow-Ups (n=474)**	**1 Follow-Up attended (n=203)**	**2 Follow-Ups attended (n=301)**	**3 Follow-Ups attended (n=255)**
Demographics										
Age (years) [mean (SD)]	75.67 (5.08)	77.46 (5.32)	75.78 (4.86)	74.83 (4.60)	73.09 (3.84)	74.40 (4.89)	75.75 (5.44)	74.94 (4.74)	73.99 (4.36)	71.95 (3.25)
Male, n (%)	1982 (45%)	758 (44%)	338 (43%)	502 (48%)	384 (43%)	426 (35%)	173 (36%)	79 (39%)	91 (30%)	83 (33%)
Female, n (%)	2451 (55%)	954 (56%)	456 (57%)	534 (52%)	507 (57%)	807 (65%)	301 (64%)	124 (61%)	210 (70%)	172 (67%)
Cardiovascular										
Obesity, n (%)	1393 (31%)	535 (31%)	256 (32%)	316 (31%)	286 (32%)	562 (46%)	200 (42%)	98 (48%)	141 (47%)	123 (48%)
Diabetes, n (%)	1099 (25%)	505 (30%)	205 (26%)	209 (20%)	180 (20%)	467 (38%)	197 (42%)	75 (38%)	111 (38%)	84 (33%)
Hypertension, n (%)	3098 (71%)	1271 (76%)	564 (72%)	681 (66%)	582 (66%)	1069 (87%)	415 (88%)	181 (89%)	258 (86%)	215 (84%)
Current Smokers, n(%)	240 (5%)	101 (6%)	45 (6%)	59 (6%)	35 (4%)	81 (7%)	37 (8%)	14 (7%)	13 (4%)	17 (7%)
Current Drinking Status, n (%)	2560 (58%)	902 (53%)	457 (58%)	641 (62%)	560 (63%)	258 (21%)	90 (19%)	38 (19%)	63 (21%)	67 (26%)
Heart Failure, n (%)	470 (11%)	264 (15%)	87 (11%)	77 (7%)	42 (5%)	233 (19%)	123 (26%)	38 (19%)	42 (14%)	30 (12%)
Heart Disease, n (%)	745 (17%)	348 (21%)	146 (19%)	153 (15%)	98 (11%)	124 (10%)	62 (13%)	20 (10%)	27 (9%)	15 (6%)
Stroke, n (%)	145 (3%)	79 (5%)	28 (4%)	26 (3%)	12 (1%)	62 (5%)	28 (6%)	13 (6%)	17 (6%)	4 (2%)
Socioeconomic Status										
Any College Education, n (%)	1992 (45%)	689 (40%)	340 (43%)	521 (50%)	442 (50%)	531 (43%)	177 (37%)	76 (37%)	133 (44%)	145 (57%)
Median Income [mean (SD)]	50225.53 (27003.01)	44990.30 (26338.88)	49608.46 (26261.35)	54461.38 (27183.55)	55474.82 (26845.46)	33446.79 (27670.38)	28014.35 (25140.89)	36345.75 (29798.07)	31780.70 (24983.48)	42392.28 (30539.86)
Dependents > 1, n (%)	3269 (74%)	1199 (70%)	580 (73%)	806 (78%)	684 (77%)	666 (54%)	248 (52%)	119 (59%)	160 (53%)	139 (55%)
ADI National Rank [mean (SD)]	42.04 (18.57)	42.15 (18.64)	43.00 (17.90)	42.08 (19.24)	40.90 (18.22)	82.61 (19.93)	83.80 (20.46)	82.85 (18.82)	83.96 (17.88)	78.60 (21.62)
Cognition										
Cognition Score [mean (SD)]	0.30 (0.78)	-0.01 (0.81)	0.31 (0.72)	0.51 (0.67)	0.66 (0.63)	-0.69 (0.88)	-1.06 (0.84)	-0.77 (0.84)	-0.48 (0.77)	-0.17 (0.75)
Dementia, n (%)	132 (3%)	111 (6%)	13 (2%)	7 (1%)	1 (0%)	61 (5%)	53 (11%)	7 (3%)	1 (0%)	0 (0%)
Physical Performance										
Composite SPPB (0-12)	9.79 (2.19)	9.06 (2.58)	9.82 (2.07)	10.26 (1.72)	10.61 (1.40)	8.50 (2.80)	7.68 (3.11)	8.52 (2.79)	8.79 (2.48)	9.65 (1.94)
Balance (0-4)	3.59 (0.93)	3.33 (1.14)	3.65 (0.85)	3.76 (0.70)	3.85 (0.56)	3.25 (1.24)	2.97 (1.38)	3.28 (1.26)	3.33 (1.17)	3.67 (0.84)
Chair Stands (0-4)	2.55 (1.23)	2.28 (1.30)	2.51 (1.22)	2.73 (1.14)	2.91 (1.04)	1.97 (1.30)	1.72 (1.35)	2.00 (1.30)	2.07 (1.25)	2.33 (1.17)
Gait (0-4)	3.65 (0.67)	3.46 (0.80)	3.66 (0.65)	3.78 (0.52)	3.85 (0.40)	3.27 (0.92)	3.00 (1.02)	3.24 (0.93)	3.39 (0.82)	3.66 (0.61)
Gait speed (m/s) [mean (SD)]	0.96 (0.22)	0.90 (0.22)	0.95 (0.20)	1.02 (0.21)	1.04 (0.21)	0.84 (0.22)	0.78 (0.22)	0.81 (0.21)	0.87 (0.21)	0.93 (0.18)

Abbreviations: ADI, Area Deprivation Index; SD, Standard Deviation; SPPB, Short Physical Performance Battery

Note: ‘No follow-up attended’ indicates that these participants attended Visit 5 exam (2011-2013) but did not attend any follow-up visits, and ‘3 Follow-Ups attended’ indicates they attended all three follow-ups

© 2026 Shrestha S et al. *JAMA Network Open.*

eTable 3. Distribution of Select Characteristics of Study Participants at Visit 5 (2011-2013), by Race and Region

Characteristics	Total (n=5666)	Black North Carolina (n=85)	Black Mississippi (n=1125)	Black Maryland (n=14)	Black Minnesota (n=9)	White North Carolina (n=1121)	White Maryland (n=1568)	White Minnesota (n=1744)	p-value
Demographics									
Age (years) [mean (SD)]	75.39 (5.07)	74.26 (5.05)	74.38 (4.87)	77.21 (5.73)	74.00 (3.43)	75.33 (5.20)	76.04 (5.19)	75.55 (4.89)	<0.001
Male, n (%)	2408 (42%)	32 (38%)	381 (34%)	8 (57%)	5 (56%)	510 (45%)	672 (43%)	800 (46%)	<0.001
Female, n (%)	3258 (58%)	53 (62%)	744 (66%)	6 (43%)	4 (44%)	611 (55%)	896 (57%)	944 (54%)	
Cardiovascular									
Obesity, n (%)	1955 (35%)	35 (41%)	516 (46%)	5 (36%)	6 (67%)	287 (26%)	570 (36%)	536 (31%)	<0.001
Diabetes, n (%)	1566 (28%)	31 (37%)	422 (38%)	8 (57%)	6 (67%)	271 (24%)	461 (30%)	367 (21%)	<0.001
Hypertension, n (%)	4167 (74%)	73 (86%)	977 (87%)	13 (93%)	6 (75%)	740 (67%)	1150 (74%)	1208 (70%)	<0.001
Current Smokers, n (%)	321 (6%)	6 (7%)	74 (7%)	1 (7%)	0 (0%)	64 (6%)	72 (5%)	104 (6%)	0.39
Current Drinking Status, n (%)	2818 (50%)	19 (24%)	228 (20%)	7 (50%)	4 (44%)	556 (51%)	682 (44%)	1322 (76%)	<0.001
Heart Failure, n (%)	703 (12%)	12 (14%)	216 (19%)	4 (29%)	1 (11%)	103 (9%)	187 (12%)	180 (10%)	<0.001
Heart Disease, n (%)	869 (16%)	9 (11%)	114 (10%)	1 (8%)	0 (0%)	187 (17%)	284 (19%)	274 (16%)	<0.001
Stroke, n (%)	207 (4%)	4 (5%)	58 (5%)	0 (0%)	0 (0%)	38 (3%)	49 (3%)	58 (3%)	0.11
Socioeconomic Status									
Any College Education, n (%)	2523 (45%)	43 (51%)	481 (43%)	3 (21%)	4 (44%)	567 (51%)	460 (29%)	965 (55%)	<0.001
Median Income [mean (SD)]	46580.63 (28014.72)	33457.83 (22783.34)	33326.42 (28121.05)	38000.00 (25323.46)	44642.86 (13418.63)	55358.65 (28049.93)	41516.48 (25051.24)	54566.83 (26078.99)	<0.001
Dependents > 1, n (%)	3935 (69%)	49 (58%)	601 (53%)	9 (64%)	7 (78%)	840 (75%)	1099 (70%)	1330 (76%)	<0.001
ADI National Rank [mean (SD)]	50.88 (25.24)	79.01 (16.31)	83.58 (19.51)	51.21 (22.60)	43.67 (20.08)	48.55 (21.14)	42.59 (16.27)	37.36 (17.41)	<0.001
Cognition									
Cognition Score [mean (SD)]	0.09 (0.90)	-0.20 (0.77)	-0.73 (0.87)	-0.83 (0.78)	-0.07 (0.89)	0.27 (0.76)	0.12 (0.79)	0.49 (0.73)	<0.001
Dementia, n (%)	193 (3%)	1 (1%)	59 (5%)	0 (0%)	1 (11%)	28 (2%)	59 (4%)	45 (3%)	0.001
Physical Performance									
Composite SPPB (0-12)	9.51 (2.40)	8.31 (2.38)	8.52 (2.84)	8.43 (2.34)	7.89 (2.09)	9.46 (2.03)	9.76 (2.24)	10.03 (2.22)	<0.001
Balance (0-4)	3.52 (1.01)	3.59 (0.97)	3.23 (1.26)	3.29 (1.27)	2.89 (1.45)	3.73 (0.77)	3.53 (0.97)	3.56 (0.97)	<0.001
Chair Stands (0-4)	2.43 (1.27)	1.74 (1.23)	2.00 (1.31)	1.93 (0.83)	1.33 (0.87)	2.21 (1.15)	2.65 (1.18)	2.68 (1.28)	<0.001
Gait (0-4)	3.56 (0.75)	2.98 (0.86)	3.29 (0.92)	3.21 (0.97)	3.67 (0.71)	3.52 (0.71)	3.58 (0.72)	3.78 (0.56)	<0.001
Gait speed (m/sec) [mean (SD)]	0.94 (0.22)	0.74 (0.18)	0.85 (0.22)	0.84 (0.24)	0.92 (0.18)	0.87 (0.17)	0.94 (0.22)	1.04 (0.22)	<0.001

Abbreviations: ADI, Area Deprivation Index; SD, Standard Deviation; SPPB, Short Physical Performance Battery

© 2026 Shrestha S et al. *JAMA Network Open.*

eTable 4. Pairwise Absolute and Relative Differences in 10-Year Composite SPPB Declines Between Regions Within Race

Region and Race Categories	Absolute Estimates (CI)	Absolute P-Values	Relative Estimates (CI)	Relative P-Values
White participants				
White North Carolina	Reference		Reference	
White Maryland	-1.14 (-1.45, -0.83)	<0.0001	2.78 (1.98, 3.91)	<0.0001
White Minnesota	0.16 (-0.13, 0.46)	0.29	0.75 (0.43, 1.30)	0.30
White North Carolina	1.14 (0.83, 1.45)	<0.0001	0.36 (0.26, 0.50)	<0.0001
White Maryland	Reference		Reference	
White Minnesota	1.31 (0.99, 1.62)	<0.0001	0.27 (0.17, 0.43)	<0.0001
White North Carolina	-0.16 (-0.46, 0.13)	0.29	1.34 (0.77, 2.33)	0.30
White Maryland	-1.31 (-1.62, -0.99)	<0.0001	3.73 (2.31, 6.04)	<0.0001
White Minnesota	Reference		Reference	
Black participants				
Black North Carolina	Reference		Reference	
Black Mississippi	-1.72 (-2.65, -0.78)	0.0003	-	-
Black Maryland	-4.17 (-6.77, -1.57)	0.0017	-	-
Black Minnesota	-	-	-	-
Black North Carolina	-	-	-	-
Black Mississippi	Reference	-	Reference	-
Black Maryland	-2.45 (-4.92, 0.01)	0.05	2.23 (1.25, 3.96)	0.006
Black Minnesota	-	-	-	-
Black North Carolina	-	-	-	-
Black Mississippi	2.45 (-0.01, 4.92)	0.05	0.45 (0.25, 0.80)	0.006
Black Maryland	Reference		Reference	
Black Minnesota	-	-	-	-
Black North Carolina	-	-	-	-
Black Mississippi	-	-	-	-
Black Maryland	-	-	-	-
Black Minnesota	Reference		Reference	

Abbreviations: SPPB, Short Physical Performance Battery; CI, Confidence Interval

Note: Models were adjusted for age, sex, obesity, diabetes, hypertension, smoking status, heart failure, coronary heart disease, stroke, education, median income, number of dependents, area deprivation index (ADI) national rank, global cognition factor score, and dementia

'-' Unstable estimates with the confidence intervals width 10 times greater than the estimate (as ascertained by (upper-lower)/estimate)) are not shown.

© 2026 Shrestha S et al. *JAMA Network Open.*

eFigure 1. Trajectories of the Composite SPPB Score Over Time by Race in the ARIC Study

Abbreviation: SPPB, Short Physical Performance Battery

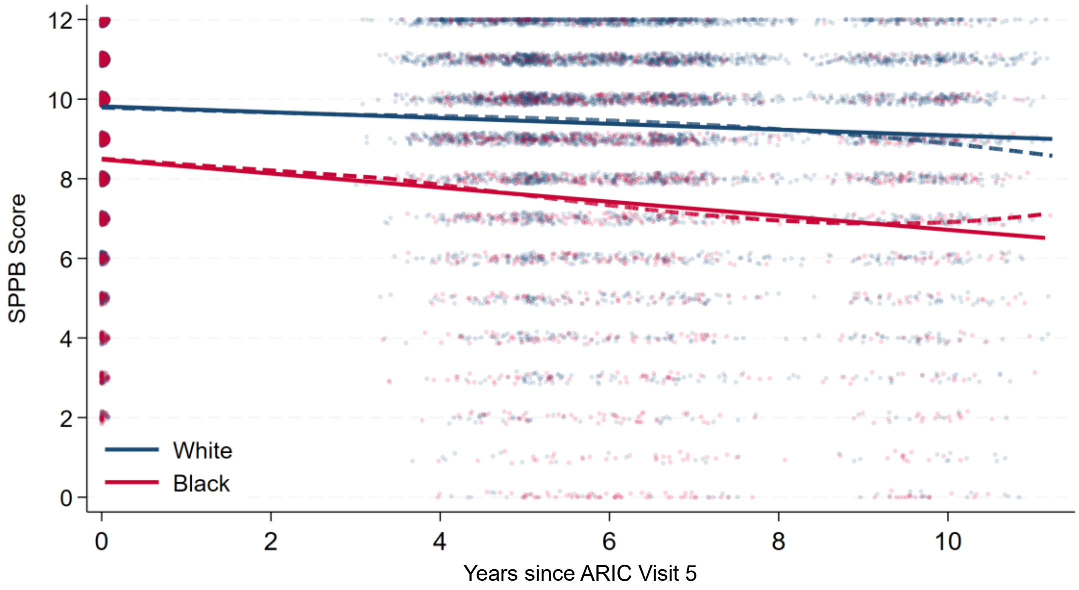

© 2026 Shrestha S et al. *JAMA Network Open.*

eFigure 2. Absolute and Relative Differences in the 10-Year Composite SPPB Declines Between Black and White Participants in the ARIC Study Applying Inverse Probability Weights for Attrition

	Absolute	Relative
**M1: Demographics**		
SPPB (0-12)	-1.48 (-1.85,-1.11)	2.13 (1.77,2.48)
SPPB Chair (0-4)	-0.62 (-0.76,-0.49)	2.06 (1.75,2.37)
SPPB Balance (0-4)	-0.33 (-0.51,-0.15)	1.64 (1.25,2.03)
SPPB Gait (0-4)	-0.49 (-0.64,-0.34)	3.12 (2.21,4.03)
Gait Speed (m/s)	-0.01 (-0.03, 0.01)	1.06 (0.93,1.18)
**M2: M1 + Cardiovascular**		
SPPB (0-12)	-1.31 (-1.71,-0.91)	1.99 (1.62,2.36)
SPPB Chair (0-4)	-0.62 (-0.77,-0.47)	2.05 (1.71,2.38)
SPPB Balance (0-4)	-0.24 (-0.43,-0.04)	1.44 (1.05,1.83)
SPPB Gait (0-4)	-0.47 (-0.63,-0.31)	3.13 (2.09,4.16)
Gait Speed (m/s)	-0.02 (-0.04, 0.00)	1.13 (0.98,1.27)
**M3: M2 + Social**		
SPPB (0-12)	-1.08 (-1.52,-0.64)	1.77 (1.41,2.14)
SPPB Chair (0-4)	-0.55 (-0.73,-0.36)	1.87 (1.51,2.23)
SPPB Balance (0-4)	-0.16 (-0.38, 0.06)	1.28 (0.87,1.70)
SPPB Gait (0-4)	-0.45 (-0.63,-0.26)	2.95 (1.83,4.07)
Gait Speed (m/s)	-0.04 (-0.07,-0.01)	1.29 (1.08,1.49)
**M4: M3 + Cognitive**		
SPPB (0-12)	-0.94 (-1.41,-0.47)	1.68 (1.29,2.07)
SPPB Chair (0-4)	-0.57 (-0.78,-0.37)	1.93 (1.52,2.34)
SPPB Balance (0-4)	-0.04 (-0.29, 0.21)	1.08 (0.63,1.52)
SPPB Gait (0-4)	-0.39 (-0.58,-0.20)	2.67 (1.56,3.77)
Gait Speed (m/s)	-0.06 (-0.09,-0.03)	1.40 (1.15,1.64)

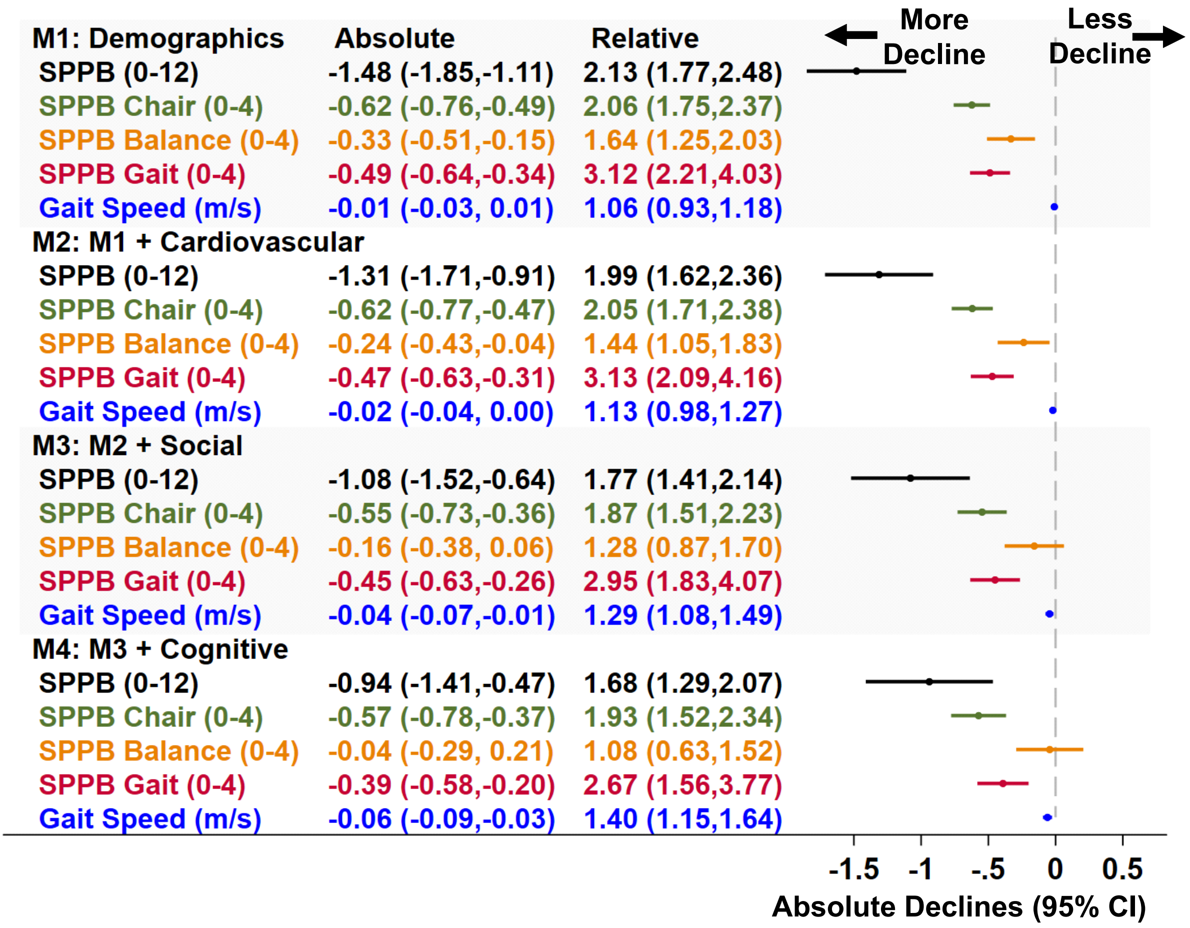

M1 (Model 1): Adjusted for age and sex. M2 (Model 2): Adjusted for Model 1 covariates and cardiovascular factors (obesity, diabetes, hypertension, cigarette smoking, alcohol drinking, heart failure, coronary heart disease, and stroke). M3 (Model 3): Adjusted for Model 2 covariates and socioeconomic status (education, median income, number of dependents, and the national Area Deprivation Index). M4 (Model 4): Adjusted for Model 3 covariates and cognitive factors (global cognitive factor score and dementia).
Abbreviations: SPPB, Short Physical Performance Battery; CI, Confidence Interval

© 2026 Shrestha S et al. *JAMA Network Open.*

eFigure 3. Comparisons of the Composite SPPB Score Differences at Visit 5 (Baseline) by Race and Region in the ARIC Study

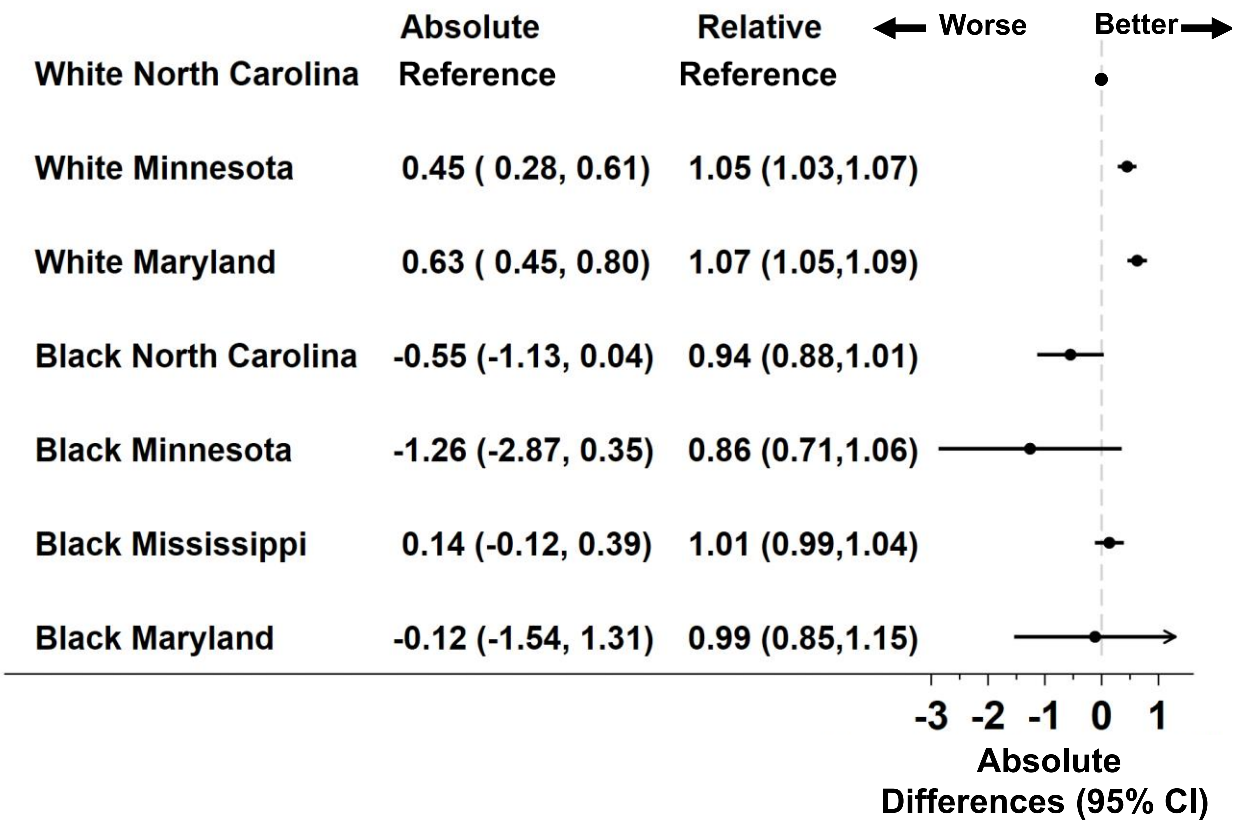

The model was adjusted for age, sex, obesity, diabetes, hypertension, smoking status, heart failure, coronary heart disease, stroke, education, median income, number of dependents, area deprivation index (ADI) national rank, global cognition factor score, and dementia. Figure shows absolute decline differences compared to White North Carolina participants (referent) with 95% Confidence Intervals by race and region. The ‘absolute’ column shows ‘absolute differences’ and the column ‘relative’ shows ‘relative differences’ in 10-year decline in composite SPPB score.

eFigure 4. Comparisons of the 10-Year Decline in the Composite SPPB Score by Race and Region in the ARIC Study Incorporating Inverse Probability Weights for Attrition

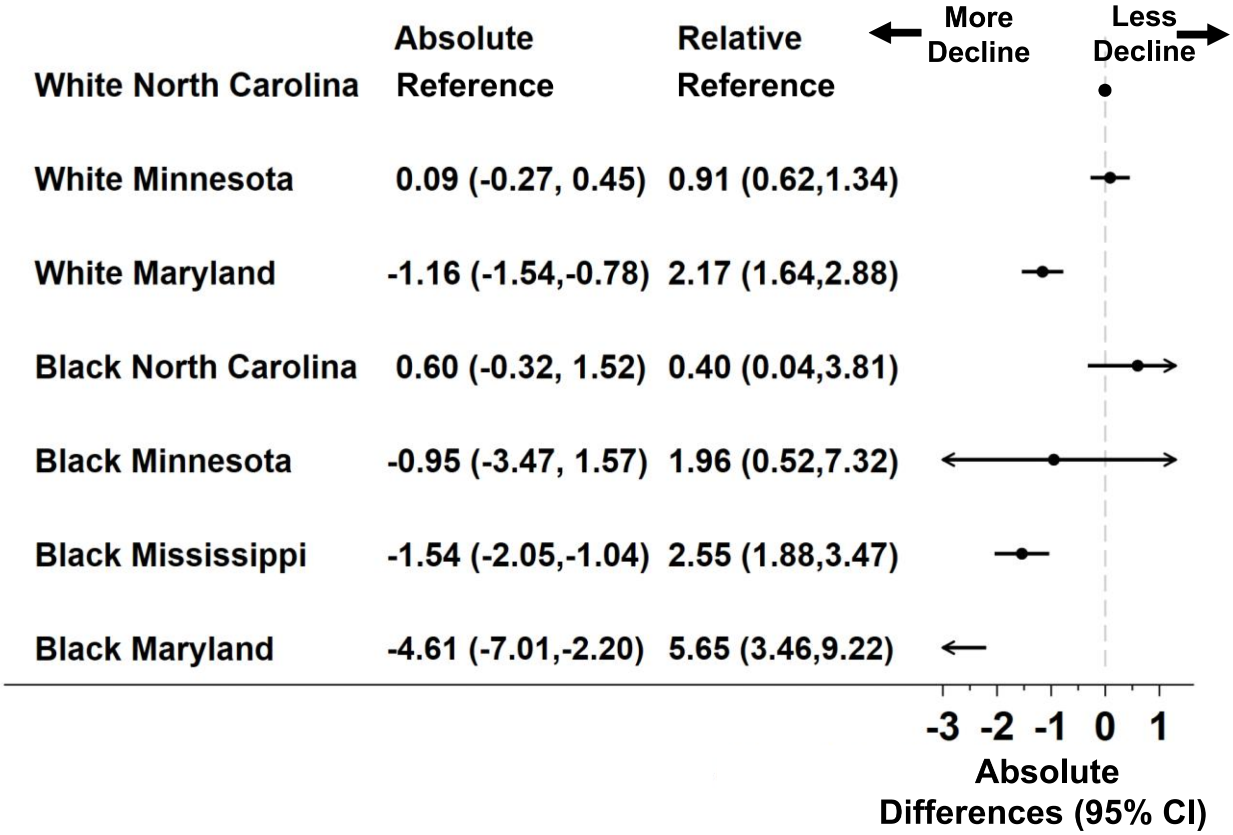

The model was adjusted for age, sex, obesity, diabetes, hypertension, smoking status, heart failure, coronary heart disease, stroke, education, median income, number of dependents, area deprivation index (ADI) national rank, global cognition factor score, and dementia. Figure shows absolute decline differences compared to White North Carolina participants (referent) with 95% Confidence Intervals by race and region. The 'absolute' column shows 'absolute differences' and the column 'relative' shows 'relative differences' in 10-year decline in composite SPPB score.

© 2026 Shrestha S et al. *JAMA Network Open.*
