# Supplementary material for: Physical Performance Changes Across Race and Region Among Black and White Older Adults
Source: JAMA Netw Open. 2026 Apr 30;9(4):e269937. doi: 10.1001/jamanetworkopen.2026.9937 (PMC13133694; doi:10.1001/jamanetworkopen.2026.9937)
Supplement: Supplement 2. — Data Sharing Statement [file jamanetwopen-e269937-s002.pdf]

Data Sharing Statement

Shrestha. Physical Performance Changes Across Race and Region Among Black and White Older Adults. *JAMA Netw Open*. Published April 30, 2026.
doi:10.1001/jamanetworkopen.2026.9937

Data
Data available: Yes
Data types: Deidentified participant data
How to access data: https://biolincc.nhlbi.nih.gov/studies/aric/
When available: With publication

Supporting Documents
Document types: None

Additional Information
Who can access the data: BioLINCC data are publicly available.
Types of analyses: Analyses may be conducted per BioLINCC requirements.
Mechanisms of data availability: BioLINCC data are publicly available without investigator support or an approved proposed. The latter can be pursued per ARIC policy, found at https://www5.cscc.unc.edu/aric9/publications/policies_forms_and_guidelines.
